# Molecular mechanisms of COVID-19-induced pulmonary fibrosis and epithelial-mesenchymal transition

**DOI:** 10.3389/fphar.2023.1218059

**Published:** 2023-08-03

**Authors:** Peng Pi, Zhipeng Zeng, Liqing Zeng, Bing Han, Xizhe Bai, Shousheng Xu

**Affiliations:** ^1^ School of Sports Medicine and Rehabilitation, Beijing Sport University, Beijing, China; ^2^ College of Physical Education and Health, East China Normal University, Shanghai, China; ^3^ School of Sports Engineering, Beijing Sport University, Beijing, China

**Keywords:** COVID-19, SARS-CoV-2, pulmonary fibrosis, epithelial-mesenchymal transition, molecular mechanisms

## Abstract

As the outbreak of COVID-19 caused by severe acute respiratory syndrome coronavirus 2 (SARS-CoV-2) first broke out in Hubei Province, China, at the end of 2019. It has brought great challenges and harms to global public health. SARS-CoV-2 mainly affects the lungs and is mainly manifested as pulmonary disease. However, one of the biggest crises arises from the emergence of COVID-19-induced fibrosis. At present, there are still many questions about how COVID-19 induced pulmonary fibrosis (PF) occurs and how to treat and regulate its long-term effects. In addition, as an important process of fibrosis, the effect of COVID-19 on epithelial-mesenchymal transition (EMT) may be an important factor driving PF. This review summarizes the main pathogenesis and treatment mechanisms of COVID-19 related to PF. Starting with the basic mechanisms of PF, such as EMT, transforming growth factor-β (TGF-β), fibroblasts and myofibroblasts, inflammation, macrophages, innate lymphoid cells, matrix metalloproteinases and tissue inhibitors of metalloproteinases, hedgehog pathway as well as Notch signaling. Further, we highlight the importance of COVID-19-induced EMT in the process of PF and provide an overview of the related molecular mechanisms, which will facilitate future research to propose new clinical therapeutic solutions for the treatment of COVID-19-induced PF.

## 1 Introduction

At the end of 2019, an unprecedented outbreak of coronavirus disease 2019 (COVID-19) caused by severe acute respiratory syndrome coronavirus 2 (SARS-CoV-2) broke out in Wuhan, Hubei Province, China, and then spread rapidly throughout China and the world (Shmakova et al., 2023). The unprecedented spread of COVID-19 has brought great challenges and harms to global public health, and attracted the attention and response of the world. According to the World Health Organization (WHO) report (https://covid19.who.int/), in early 2023, there were more than 750 million confirmed cases of COVID-19 worldwide, including more than 6.8 million deaths. SARS-CoV-2 spreads rapidly among humans primarily through respiratory droplets when an infected person coughs, sneezes, and speaks. The process of SARS-CoV-2 infection is mainly through the interaction between the angiotensin-converting enzyme 2 (ACE2) receptor and transmembrane serine protease 2 (TMPRSS2) on the surface of host cells (Hoffmann et al., 2020). In detail, SARS-CoV-2 infects cells lining the air passageways by locking the spike protein to the ACE 2 receptor and replicates both in upper and lower respiratory tract (Harrison et al., 2020; Nieman, 2020). Depending on the severity of the disease, SARS-CoV-2 infection can be asymptomatic or can range from mild fever to life-threatening complications (Jamil et al., 2020; Safiabadi et al., 2021). Infection with the virus mainly causes fever, inflammation, and even severe acute respiratory distress syndrome (ARDS), accompanied by symptoms such as cough, fatigue, muscle weakness, dyspnea, diarrhea, nausea and vomiting (Garg et al., 2020; George et al., 2020; Wu et al., 2020).

The lung is the main organ of SARS-CoV-2 infection, its main manifestation are pulmonary diseases. In addition, organs such as kidney and liver may also be infected by the virus (Kolesova et al., 2021; Legrand et al., 2021; Jansen et al., 2022; Saifi et al., 2022). However, COVID-19-induced fibrosis is one of the most serious harms arising from viral infection (Wendisch et al., 2021). It has been shown that SARS-CoV-2 infection leads to the upregulation of mRNA levels of certain fibrosis drivers in humans (e.g., ACE 2), which is associated with a decrease in epithelial-mesenchymal transition (EMT), cell proliferation, stemness, and downregulation of oncogenic pathways (Xu J. et al., 2020; Zhang C. et al., 2020). Approximately 30 percent of patients present with SARS-CoV-2-associated pulmonary fibrosis (PF), and this proportion increased with disease severity and duration. Established fibrosis worsened already impaired lung function in COVID-19 survivors (Francone et al., 2020; Fu et al., 2020; Vasarmidi et al., 2020; Bocchino et al., 2022; Saifi et al., 2022; Shmakova et al., 2023). Even after recovery from COVID-19, up to 17% of patients will develop PF (Pan et al., 2020). As an important process of fibrosis, EMT may be the basis of the mechanism of PF in COVID-19 patients (Zhang et al., 2022). Recent evidence revealed that SARS-CoV-2 directly infects renal cells, such as podocytes and tubular epithelium, which are associated with kidney injury and fibrosis (Braun et al., 2020; Bowe et al., 2021). At present, the treatment methods for PF in COVID-19 patients are very limited. Therefore, the aim of this study is to review the molecular mechanisms of COVID-19-induced PF and EMT, and provide a theoretical basis for promoting the health management of PF in COVID-19 patients.

## 2 Basic mechanisms of PF

### 2.1 EMT

EMT is a dynamic and reversible transdifferentiation process in which epithelial cells lose part of their epithelial cell characteristics and acquire the typical characteristics of mesenchymal cells (Inui et al., 2021). As one of the fundamental processes that are activated in the primary stages of life, three types of EMT can produce different functional consequences. Among them, type II EMT may lead to certain pathophysiological conditions, including fibrosis and organ destruction (Acloque et al., 2009; Lee et al., 2021; Saifi et al., 2022). Type I and type III EMT are associated with development and cancer, respectively. The main characteristics of PF are alveolar epithelial cell damage, fibroblast overproliferation, and increased extracellular matrix protein deposition (Zhang et al., 2019; Lee et al., 2021). It is an interstitial lung disease that predisposes to scarring in the lung and irreversible decline in lung function (Herrera et al., 2018). Lung epithelial cell injury is an important event in this process. Specifically, after the production of its injury, EMT is activated and generates fibroblast-like cells, induces abnormal increase and excessive deposition of myofibroblasts and extracellular matrix (ECM) as well as remodeling of the pulmonary interstitium (Pozharskaya et al., 2009; Richeldi et al., 2017; Yue et al., 2023). About one-third of the fibroblast-like cells are derived from the epithelium (Peng L. et al., 2020). Furthermore, transforming growth factor-β (TGF-β) is one of the major drivers of EMT in PF and it has been determined that its level is increased in damaged lung epithelial cells (Willis and Borok, 2007).

### 2.2 Role of TGF-β

TGF-β is a multifunctional cytokine that can regulate a wide range of physiological processes. Specifically, TGF-β is a key mediator in the pathogenesis of almost all fibrosis, including PF (Bergantini et al., 2022). It is mainly involved in the activation, proliferation and differentiation of epithelial cells and collagen-producing myofibroblasts (Wu et al., 2021). TGF-β induces excessive fibroblast proliferation and differentiation into myofibroblasts, a central pathway in PF that is capable of leading to increased production and abnormal deposition of ECM (Delpino and Quarleri, 2020). In addition, TGF-β is capable of triggering the overexpression of profibrotic genes by activating different signals (Weiss and Attisano, 2013; Hu et al., 2018; Lee et al., 2021). A lot of evidence suggested that TGF-β signaling is critical for the induction of EMT (Heldin and Moustakas, 2016; Nieto et al., 2016; Hao et al., 2019). The main factors regulating TGF-β signaling include SNAIL, zinc-finger E-box-binding (ZEB), α-smooth muscle actin (α-SMA), TWIST and matrix metalloproteases (MMPs) (Hao et al., 2019; Lee et al., 2021). In addition, SNAIL activated vimentin, and N-cadherin also play an important role in regulating TGF-β signaling (Cano et al., 2000; Moreno-Bueno et al., 2006). The major signaling pathways that promote TGF-β-induced EMT are mediated by both SMAD-dependent and SMAD-independent pathways (Fernandez and Eickelberg, 2012; Park et al., 2021). Specifically, SMAD-dependent signaling mainly induces the expression of α-SMA, collagen, plasminogen activator inhibitor-1 (PAI-1) and connective tissue growth factor (CTGF). Meanwhile, SMAD-dependent AKT activation leads to nuclear translocation of β-catenin, resulting in upregulation of α-SMA; by contrast, SMAD-independent signaling leads to loss of tight junctions, rearrangement of cytoskeletal structures and nuclear translocation of β-catenin, and ultimately increased cell mobility, through activation of partitioning-defective protein 6 (PAR6), ras homolog family member A (RhoA) and phosphoinositide 3-kinase (PI3K)/AKT pathways (Willis and Borok, 2007; Fernandez and Eickelberg, 2012). The family of secretory polypeptide growth factors, modeled on TGF-β, has been found to have more than 40 members, and these cytokines play a crucial role by controlling a wide variety of cellular proliferation and differentiation (Roberts et al., 1981). Among them, TGF-β1 has been observed to be upregulated in various fibrotic processes, it activates its downstream factors Smad 2, Smad 3, Smad 4, and Smad 7 through TGF-β receptor I (TβRI) and TβRII and plays a crucial role in EMT and fibrogenesis (Chen et al., 2018; Eser and Janne, 2018; Hu et al., 2018; Wang J. et al., 2022). In fibrosis, TGF-β1 can affect multiple pathways such as fibroblast proliferation and EMT activation (Saifi et al., 2022).

### 2.3 Fibroblasts and myofibroblasts

Fibroblasts are a kind of diverse mesenchymal cells whose main shared function is to produce connective tissue by synthesizing ECM (Plikus et al., 2021). To promote tissue repair, fibroblasts are induced to form myofibroblasts by signal and physical factors (Pakshir et al., 2019). Therefore, myofibroblasts are also referred to as activated fibroblasts. Specifically, acute injury may cause the reduction of alveolar epithelial cells, the destruction of alveolar structure, as well as the release of proinflammatory mediators, thereby increasing the upregulation of cytokines such as tumor necrosis factor α (TNF-α), TGF-β, interleukin-1β (IL-1β), and IL-6 (Ramachandran et al., 2015; Chen et al., 2019; Zhao et al., 2022). Subsequently, pulmonary fibroblasts are activated to form myofibroblasts through the upregulation of fibrotic cytokines such as platelet-derived growth factor (PDGF), fibroblast growth factor (FGF), and vascular endothelial growth factor (VEGF) (Fan et al., 2012; Frangogiannis, 2014; Zhao et al., 2022). When pulmonary fibroblasts and myofibroblasts overproduce and deposit ECM under the influence of chronic injury or persistent inflammation, it leads to reduced gas exchange and impaired lung function, resulting in local scarring or diffuse, idiopathic pulmonary fibrosis (IPF) (Desmouliere et al., 2005; Shi-wen et al., 2009; Chanda et al., 2019). Hyperproliferation of myofibroblasts can also be seen in severe COVID-19 patients (Delorey et al., 2021; Wang et al., 2021). Therefore, reducing the myofibroblast population or inhibiting its activation is beneficial to slow the progression of fibrosis in these patients.

### 2.4 Inflammation

As we know, inflammation is a component of fibrosis process, and PF is a chronic inflammatory disease of lung tissue. In addition to EMT, inflammation is causally related to the formation of fibrosis. Specifically, injury to lung tissue is often accompanied by inflammation, activation of which contributes to up-regulating inflammatory mediators and promotes the recruitment of neutrophils, eosinophils, and macrophages to the injured site to clear debris and necrotic areas while releasing profibrotic mediators. Pulmonary fibroblasts are then activated into myofibroblasts by upregulation of fibroblastic factors such as FGFs, PDGFs, and VEGFs. Myofibroblasts are abnormally and constantly activated and secrete ECM components, which eventually leads to excessive deposition of ECM and formation of PF (Kishore and Petrek, 2021; Zhao et al., 2022) and thus, the inflammatory response plays a complex and crucial role in the development of PF (Peng Y. et al., 2020; Kishore and Petrek, 2021; Saifi et al., 2022). After SARS-CoV-2 infection, the sudden release of proinflammatory cytokines in the circulation activates an excessive immune response, which leads to increased recruitment of inflammatory cells, including macrophages and monocytes (Hirawat et al., 2021). This causes a lot of cytokines, such as IL-2, IL-7, and interferon γ (INF-γ)-induced protein-10 (IP-10), to be released in patients with COVID-19. Evidence has shown that a large number of pulmonary infiltrates (neutrophils and macrophages) and the subsequent inflammatory cytokine storm in the COVID-19 pandemic are closely related to lung injury, ARDS and other secondary complications (Mehta et al., 2020; Zhou et al., 2020; Barriga et al., 2021). In addition, the inflammasome is a large intracellular multiprotein complex and a special inflammatory signal transduction platform, which plays an important role in responding to pathogen-associated molecular patterns (PAMPs) and damage-associated molecular patterns (DAMPs) (Lee J. et al., 2016; Moossavi et al., 2018). Nucleotide-binding oligomerization domain-like receptor family pyrin domain-containing 3 (NLRP3) inflammasome is currently the most representative inflammasome (Schroder and Tschopp, 2010; Strowig et al., 2012). When PAMPs or DAMPs are recognized, the nucleus factor-κB (NF-κB) signaling pathway is activated, thereby up-regulating the transcription of NLRP3, pro-IL-1β, pro-IL-18 and other inflammasome related components. The inflammasome adaptor protein, apoptosis-associated speck-like protein containing caspase-recruitment domain (ASC) is then recruited to NLRP3 to interact with caspase-1, which results in the activation of NLRP3 inflammasome. Recent studies have shown the regulatory role of NLRP3 inflammasome in a variety of pulmonary diseases such as PF (Peng L. et al., 2020). It has been reported that in a bleomycin (BLM)-induced model of PF, NLRP3 −/− mice show reduced neutrophil influx and IL-1β levels in the lung (Lee S. et al., 2016). Additionally, NLRP3 inflammasome was also shown to be involved in the regulation of EMT in BLM-induced PF (Tian et al., 2017). These evidences indicate that NLRP3 inflammasome is involved in the pathogenesis of PF, suggesting that inhibiting EMT process or anti-inflammation by regulating NLRP3 inflammasome is a potential therapeutic strategy for PF. Furthermore, some evidence suggests that SARS-CoV-2 may directly activate the NLRP3 inflammasome, leading to the activation of macrophages, neutrophil infiltration, and excessive production of cytokines, which eventually cause cytokine storm and fibrosis (van den Berg and Te, 2020). Overall, SARS-CoV-2 infection-associated NLRP3 inflammasome activation may contribute to pulmonary inflammation and fibrosis (Effendi and Nagano, 2021a).

### 2.5 Role of macrophages

Since M2 type macrophages are able to activate TGF-β/Smad and IL-6/signal transducer and activator of transcription 3 (STAT3) signaling pathways, macrophages are considered to be a key factor in the development of PF (Wang Y. et al., 2022). When the body produces injury, macrophages rapidly arrive and promote wound healing and repair (Saifi et al., 2022). Macrophages are involved in the whole process of lung injury and repair, which can promote and inhibit PF (Liu et al., 2019; Goda et al., 2020). Alveolar macrophages (AM) and interstitial macrophages (IM) are two distinct populations of macrophages that maintain pulmonary homeostasis (Byrne et al., 2016; Kishore and Petrek, 2021). M1 and M2 macrophages are different cell phenotypes of AM and IM respectively polarized during the process of tissue injury and inflammation developing into PF (Zhang et al., 2018; Liu et al., 2019; Kishore and Petrek, 2021). When the polarization of M2 macrophages is enhanced, it can regulate the development of fibrotic lung disease or inhibit inflammatory response by producing chemokines, MMPs, and fibronectin (Polverino et al., 2020; Kishore and Petrek, 2021). On the other hand, the transition from M1 to M2 macrophages induces fibrosis by releasing certain profibrotic mediators such as TGF-β, insulin-like growth factor-1 (IGF-1), FGF-2, PDGF (Hou et al., 2018). Thus, the proportion of M2 macrophages is higher in most types of interstitial lung disease, including IPF (Kishore and Petrek, 2021). Recently, it has been proposed that COVID-19-associated macrophages are significantly similar to the profibrotic macrophage populations found in IPF (Wendisch et al., 2021). Therefore, the mechanism by which the response of macrophages affects PF after SARS-CoV-2 infection deserves further investigation.

### 2.6 Innate lymphoid cells

Innate lymphoid cells (ILCs) are one of the important subsets of innate immune cells, including natural killer (NK), innate lymphocyte-1 (ILC1s), ILC2s and ILC3s cells. ILCs play a crucial role in tissue repair and homeostasis, regulating protective immunity in various mucosal tissues (Ardain et al., 2019; Deng et al., 2023). Among the many factors that constitute the innate immune system, the types of inflammatory process promoted by ILCs and the expression of their cytokines suggest that they are important contributors to the pathogenic process of fibrosis (Zhang et al., 2016; Ardain et al., 2019). NK cells and ILC1 cells are considered to be the same group of ILCs due to their secretion of IFN-γ and TNF-α (Deng et al., 2023). Depletion of CXCR3 has been reported to impede NK cell recruitment to the lung and subsequent IFN-γ production promotes PF in a mouse model of BLM-induced PF, suggesting that NK cell activation may regulate the development of PF through IFN-γ production (Jiang et al., 2004). Furthermore, previous studies have shown that T helper 2 cell (Th-2) cytokines IL-4, IL-5, and IL-13 are involved in inflammation and tissues fibrosis in the lung, and ILC2 also participates in these processes by directly producing Th2 cytokines and assisting Th2 activation via major histocompatibility complex classes II (MHCII) and IL-13 (Neill et al., 2010; Duffield et al., 2013; Wick et al., 2013). It has been found that the levels of IL-25 and ILC2s are increased in the bronchoalvoelar lavage and lung tissues of patients with IPF (Hams et al., 2014). Moreover, dermal and circulating ILC2 counts are closely associated with the development of PF in patients with systemic sclerosis, suggesting that ILC2s may exacerbate PF in these patients. In addition, Li et al. proposed that IL-33 activates M2 macrophages to produce IL-13 and TGF-β1, which in turn induces ILC2 expansion to produce IL-13, ultimately leading to the development of PF (Li et al., 2014). It is worth emphasizing that even though ILC3s are fewer in number, they are able to produce large amounts of IL-17, IL-22, and pulmonary cytokines such as granulocyte-macrophage colony-stimulating factor (GM-CSF), possessing the potential to drive inflammation and affect lung health (Deng et al., 2023).

### 2.7 MMPs and tissue inhibitors of metalloproteinases

MMPs are zinc-dependent endopeptidases, belonging to the extracellular endopeptidases family, which are mainly involved in the degradation of ECM substrates such as collagen, fibronectin, and laminin, and contribute crucially to maintaining the homeostasis of ECM (Chuliá-Peris et al., 2022). There are 25 members of this family with variable effects on fibrotic ECM. Some MMPs are profibrotic, primarily by stimulating EMT, while others are the opposite (Mahalanobish et al., 2020). The growth factors, inflammatory mediators and receptors released by MMPs from the ECM can regulate tissue repair, immune response, apoptosis and proliferation, which are the main pathological mechanisms involved in PF and play a critical role in its development (Cui et al., 2017). Furthermore, the mechanism by which MMPs affect PF also includes that MMP-3 and MMP-9 can promote abnormal repair processes, such as abnormal epithelial cell migration; MMP-3, MMP-7, and MMP-8 can increase/decrease the pulmonary levels or activities of profibrotic/antifibrotic mediators. MMP-8 and MMP-10 can promote the transformation of pulmonary macrophage M1 into M2 subtype (Chuliá-Peris et al., 2022). On the other hand, tissue inhibitors of metalloproteinases (TIMPs) are endogenous protein inhibitors of MMPs, consisting of four family members (TIMP 1–4), which reversibly inhibit the activity of MMPs by noncovalently complexing with them at a 1:1 molar ratio (Majali-Martinez et al., 2016; Mahalanobish et al., 2020). Hence, the ultimate accumulation or degradation of ECM is the result of differential expression of MMPs *versus* TIMPs.

### 2.8 Hedgehog pathway

The Hedgehog (Hh) pathway is a complex cell signaling pathway that is essential for cell differentiation and proliferation, coordination of growth factors and transcription factors, as well as tissue structure formation during normal embryonic development. It is also involved in organogenesis, homeostasis and regeneration. Notably, recent studies have shown that Hh activation can promote the development of fibrosis by inducing the transformation of fibroblasts into myofibroblasts (Effendi and Nagano, 2021b). As one of the three orthologs of Hh, sonic hedgehog (Shh) signaling is one of the most important signaling pathways in the formation of PF (Chanda et al., 2019). Mechanistically, Shh promotes macrophage secretion of osteopontin (OPN) through the Shh/glioma-associated onco-gene (Gli) signaling pathway, which is a multifunctional glycoprotein involved in a variety of physiological and pathological processes, including chronic inflammation and tissue remodeling. Secreted OPN acts on peripheral macrophages in an autocrine or paracrine manner and induces macrophage alternative polarization and PF by activating the Janus kinase 2 (JAK 2)/STAT 3 signaling pathway (Hou et al., 2021).

### 2.9 Notch signaling

Notch is an important signaling pathway with four transmembrane receptors (Notch 1–4) and five ligands, referred to as Jagged 1 (JAG1), JAG2, delta-like 1 (Dll1), Dll3, and Dll4 in mammals (Saifi et al., 2022). The normal conduction of Notch signaling can promote the development and homeostasis of multiple organs, while the dysregulation of Notch signaling is often associated with tissue fibrosis (Chanda et al., 2019). Activation of Notch signaling is able to promote myofibroblast proliferation and differentiation in fibrosis of several organs, including the lung (Vera et al., 2021). Additionally, Notch signaling is capable of activating EMT, inducing a transition of the epithelial phenotype to the mesenchymal subtype and further aggravating fibrosis (Gopalakrishnan et al., 2014; Saifi et al., 2022). Studies have shown that Notch 1 is activated in the early stage of IPF and is an important regulator of alveolar epithelial type 2 cells (AEC2). Its main role is to induce alveolar epithelial proliferation and promote fibroproliferation, resulting in PF (Wasnick et al., 2023). Furthermore, JAG1 ligands play a crucial role in TGF-β-induced PF, as RNA silencing of JAG1 or chemical inactivation of Notch is capable of blocking TGF-β-induced EMT (Zhao et al., 2018).

**FIGURE 1:**
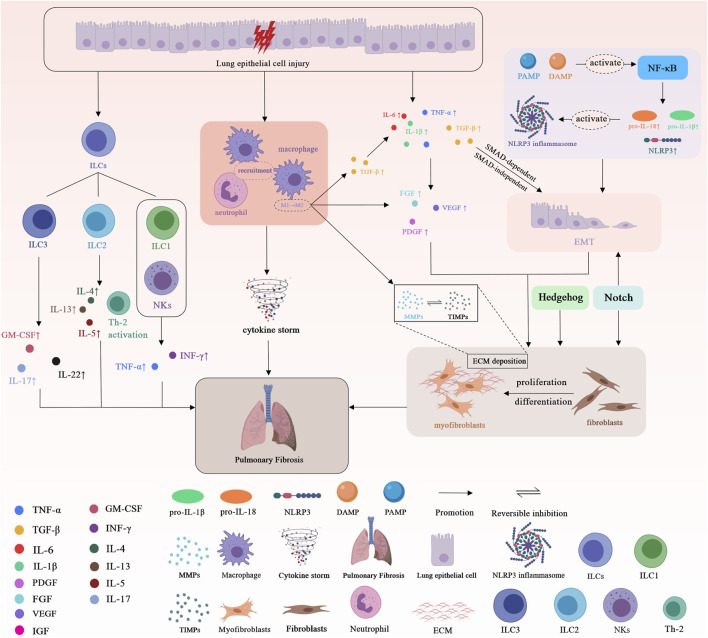
Schematic representation of the basic underlying mechanisms of pulmonary fibrosis. Tumor necrosis factor α (TNF-α); Transforming growth factor-β (TGF-β); Interleukin-6 (IL-6); Interleukin-1β (IL-1β); Interleukin-18 (IL-18); Interleukin-4 (IL-4); Interleukin-5 (IL-5); Interleukin-13 (IL-13); Interleukin-17 (IL-17); Interleukin-22 (IL-22); Granulocyte-macrophage colony-stimulating factor (GM-CSF); Interferon γ (INF-γ); Platelet-derived growth factor (PDGF); Fibroblast growth factor (FGF); Vascular endothelial growth factor (VEGF); Insulin-like growth factor-1 (IGF-1); Matrix metalloproteases (MMPs); Tissue inhibitors of metalloproteinases (TIMPs); Extracellular matrix (ECM). Epithelial-mesenchymal transition (EMT); Pathogen-associated molecular pattern (PAMP); Damage-associated molecular pattern (DAMP); Nucleus factor-κB (NF-κB); Nucleotide-binding oligomerization domain-like receptor family pyrin domain-containing 3 (NLRP3); Innate lymphoid cells (ILCs); Natural killer cells (NKs); T helper 2 cell (Th-2).

Overall, the common underlying mechanisms of PF are shown in Figure 1.

## 3 Molecular mechanisms of COVID-19-induced EMT

### 3.1 SARS-CoV-2 directly induces EMT

The mechanisms and association between EMT and PF have been described previously. Interestingly, some studies have proposed that SARS-CoV-2 can induce EMT in lung cancer cells, and EMT signals in the upper respiratory tract were detected in the nasal epithelia of COVID-19 patients, and infection both *in vivo* and *in vitro* may increase the expression of EMT-related genes (Downes et al., 2021; Pandolfi et al., 2021; Stewart et al., 2021). Recently, one study found that SARS-CoV-2 infection of lung cancer cells led to upregulation of ZEB1 and anexelekto (AXL) mRNA levels, which induced EMT. Specifically, ACE 2 receptor functions were completely inhibited by overexpression of ZEB1 and TGF-β. ZEB1 inhibits ACE2 by two repressor binding sites within the ACE2 promoter, and forced overexpression of ZEB1 leads to a significant reduction in ACE2 expression (Stewart et al., 2021). These findings indicated that COVID-19 infection significantly induced EMT (Sinha and Kundu, 2021; Stewart et al., 2021). Besides, when serum samples from COVID-19 patients were applied to cultures of different cancer cells, the cancer cells showed increased motility and loss of intercellular junctions. Gene expression analysis showed that ZEB1, SNAIL2, and vimentin (VIM) mRNA levels were highly expressed, which indicated the activation of EMT (Saygideger et al., 2021; Sinha and Kundu, 2021). Furthermore, RNAseq data analysis of A549 and Calu-3 cells infected with SARS-CoV-2 for 24 h showed that epithelial cell adhesion molecules (EPCAM) gene expression was downregulated in epithelial cells, and ZEB1 was upregulated in Calu-3, A549, and A549 + ACE2 cell lines after infection. This also confirms that SARS-CoV-2 infection induced changes in epithelial phenotype (Blanco-Melo et al., 2020; Stewart et al., 2021). Similar to ZEB1, AXL is another regulator of EMT that is strongly associated with the mesenchymal phenotype, and SARS-CoV-2 infection upregulated AXL expression in the three cell lines mentioned above. Finally, COVID-19-induced EMT was also found to occur in 430 COVID-19 patients, and severe epithelial dysfunction and alveolar damage were found in 3 autopsy patients with COVID-19, accompanied by the reorganization indicative of EMT (He et al., 2020; Lieberman et al., 2020; Downes et al., 2021). Collectively, these studies suggest that SARS-CoV-2 infection increases EMT and that EMT and related proteins such as ZEB1 and AXL are novel therapeutic targets against COVID-19.

### 3.2 Neutrophils and NETosis drive EMT after SARS-CoV-2 infection

NETosis is a mechanism by which neutrophils fight against pathogens and protect the body from their invasion, which can release neutrophil extracellular traps (NETs) (Lefrançais et al., 2018; Pandolfi et al., 2021). Neutrophils are the effector cells of NETs production. NETs are reticular structures composed of chromatin modified by proteases, such as human neutrophil elastase (HNE) and myeloperoxidase (MPO), which mainly limit the spread of pathogens in body tissues (de Bont et al., 2019; Pandolfi et al., 2021). The increase in their number is positively correlated with the severity of COVID-19, and the involvement of NETs in COVID-19 is fully supported by neutrophil recruitment during SARS-CoV-2 infection of the lungs (Middleton et al., 2020). Viral infection is a prerequisite for neutrophils to induce NETosis and the release of NETs (Zhu et al., 2022). When neutrophils are fully infiltrated by SARS-CoV-2 virus, the production of NETs can be promoted, and this process is limited by classical infection mechanisms such as ACE2 and serine proteases (Veras et al., 2020). In addition, SARS-CoV-2 can also infect neutrophils through atypical entry mechanisms, such as the C-type lectin receptor (Lu et al., 2021). In fact, SARS-CoV-2 infection can induce neutrophils to increase the production of pro-NETosis mediators and thus promote the release of NETs, which is another mechanism by which SARS-CoV-2 induces NETosis and NETs (Zhu et al., 2022). Furthermore, there are other indirect mechanisms for SARS-CoV-2-induced NETosis and NETs. For example, SARS-CoV-2 induces the release of proinflammatory mediators, or DAMPs, when it infects epithelial cells or comes into contact with other neighboring cells. Specifically, a large number of proinflammatory mediators such as IL-8 and IL-1β produced by SARS-CoV-2-infected epithelial cells and macrophages are also important mediators in the induction of NETs (Park and Lee, 2020; Yaqinuddin and Kashir, 2020; Zhu et al., 2022). Excessive NETosis not only induces tissue damage but also promotes EMT. Neutrophils can effectively induce EMT through NETosis in the lungs of severe COVID-19 patients. Studies have confirmed that overexpression of the mesenchymal marker a-SMA and decreased expression of the epithelial marker E-cadherin can be observed 24 h after NETs were added to A549 cells, a common model of type II lung cells (Pandolfi et al., 2021). Thus, EMT can also be induced directly by NETs. In addition, neutrophils can also induce EMT by releasing TGF-β, neutrophil gelatinase-associated lipocalin (NGAL), or protease-activated receptor 4 (PAR 4) (Ando et al., 2007; Wang et al., 2017). Therefore, inhibition of NETs in COVID-19 may ameliorate their mediated EMT and fibrosis.

### 3.3 SARS-CoV-2 induces the upregulation of TGF-β to drive EMT

In the process of SARS-CoV-2-induced PF, the increased oxidative stress of epithelial cells promotes the production and release of TGF-β. In addition, infection with SARS-CoV-2 causes apoptosis of alveolar cells, T-lymphocytes and pneumocytes, which leads to the death of neutrophils (Sanjabi et al., 2017). To engulf and digest dead cells, macrophages enter the lungs and release more TGF-β after clearing debris (Sanjabi et al., 2017; Hirawat et al., 2023). It was reported that the expression of TGF-β is highly increased in patients with COVID-19. TGF-β stimulates alveolar macrophages by inducing the secretion of IL-4, IL-6, and IL-13, thereby promoting the development of PF (Delpino and Quarleri, 2020; Bergantini et al., 2022). After SARS-CoV-2 infection of alveolar epithelial cells, the expression of TGF-β1 mRNA transcripts was significantly increased (Xu Z. et al., 2020). In addition, TGF-β can induce the activity of many downstream signaling factors, including the PI3K/AKT, extracellular signal-regulated kinase (ERK), and Smads pathways (Guo and Wang, 2009). Among them, the activation of AKT pathway induces TGF-β-dependent EMT, which in turn leads to the upregulation of β-catenin and type II collagen (COL2A1), leading to collagen accumulation. TGF-β/Smads signaling was confirmed to regulate EMT.

### 3.4 Plasminogen activator system in COVID-19-induced EMT and PF

The relationship between plasminogen activation system and acute lung injury has been vigorously discussed. At the same time, its role in COVID-19-induced PF is also being widely studied (Shmakova et al., 2023). Studies have shown that urokinase plasminogen activator (uPA) and urokinase-type plasminogen activator receptor (uPAR) are involved in the pathogenesis of COVID-19 and contribute to the development of PF through single-cell RNA-seq and immunohistochemical analysis. uPAR is a receptor with three domains (D-I, D-II and D-III), which is usually anchored on the surface of various cells such as neutrophils and macrophages through glycosylphosphatidylinositol (GPI) (Smith and Marshall, 2010; D'Alonzo et al., 2020). Its activity may affect the pathogenesis of SARS-CoV-2 through S protein cleavage and fibrinolytic balance (Alfano et al., 2022). The major ligand of uPAR is uPA, to which uPAR binds and converts plasminogen to plasmin. Plasmin degrades the components of the ECM through a series of proteolytic cascades (D'Alonzo et al., 2020). Upregulation of uPA/uPAR system mainly involves elevated levels of proinflammatory cytokines and chemokines, proliferation and apoptosis of epithelial and endothelial cells, and remodeling of damaged tissues. A large number of previous studies have shown that the overexpression of uPAR is closely related to EMT (Alfano et al., 2022). In the recent study, it was found that the decreased expression of uPAR in lung tissues of COVID-19 patients led to the increased level of uPA, which subsequently caused the upregulation of IL-6 and ACE2 expression and significantly promoted PF by inducing EMT of lung epithelial cells. Specifically, ACE2 can enhance the interaction between SARS-CoV-2 and epithelial cells (Fagyas et al., 2022; Zheng, 2022). Both uPAR and IL-6 can regulate the process of EMT (Semina et al., 2020; Abaurrea et al., 2021). One of the major roles of uPAR is to maintain the epithelial phenotype of epithelial cells, and uPAR reduction in lung tissue triggers PF-induced EMT. In the experimental results of lung epithelial cells, downregulation of uPAR was associated with increased N-cadherin expression, which confirmed the induction of EMT. The potential of plasminogen in the treatment of COVID-19-induced PF deserves further exploration.

**FIGURE 2:**
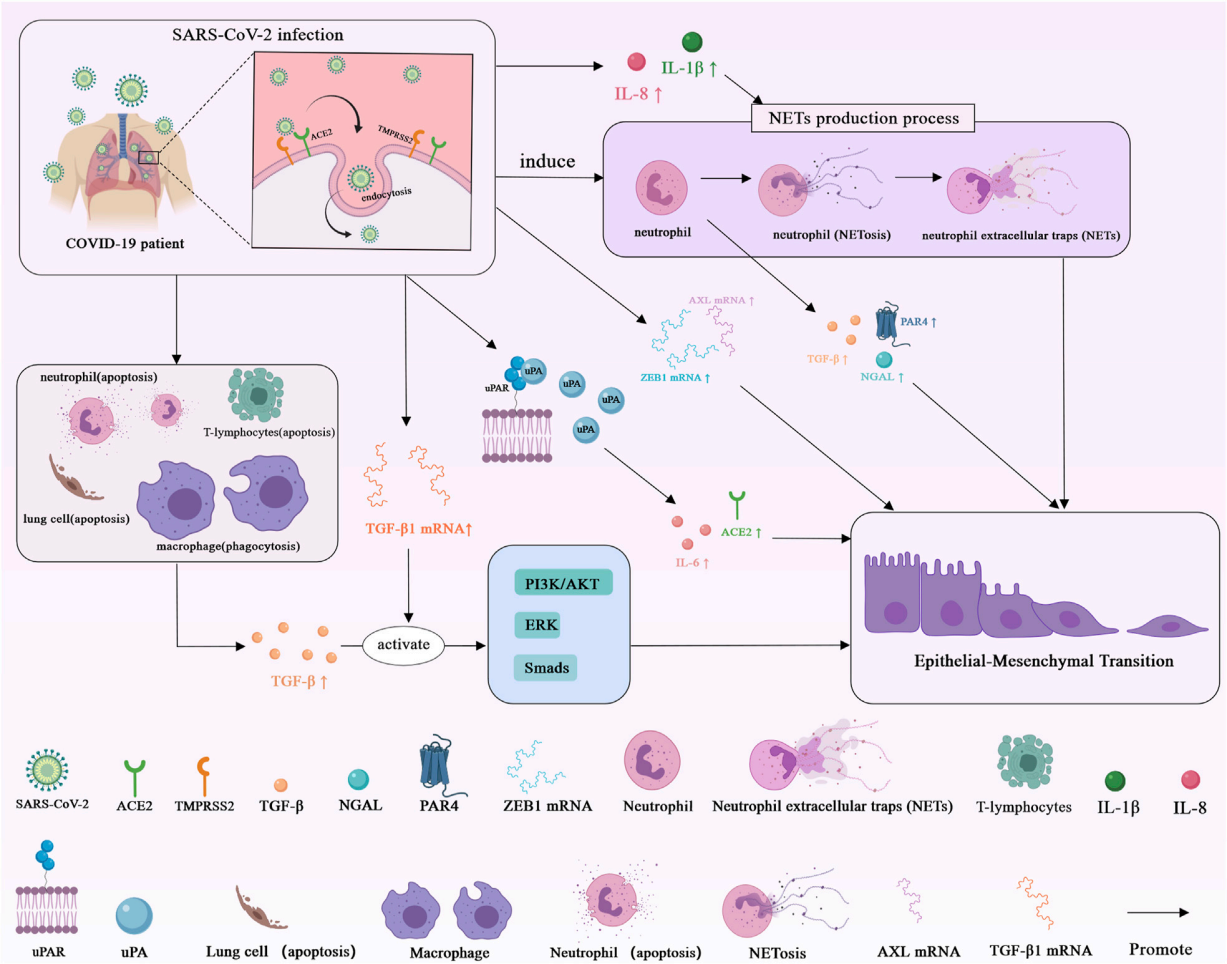
Schematic representation of the molecular mechanism of COVID-19-induced EMT. Angiotensin-converting enzyme 2 (ACE2); Transmembrane serine protease 2 (TMPRSS2); Transforming growth factor-β (TGF-β); Transforming growth factor-β1 (TGF-β1); Interleukin-8 (IL-8); Interleukin-6 (IL-6); Interleukin-1β (IL-1β); Neutrophil gelatinase-associated lipocalin (NGAL); Protease-activated receptor 4 (PAR 4); Zinc-finger E-box-binding 1 (ZEB1); Urokinase-type plasminogen activator receptor (uPAR); Urokinase plasminogen activator (uPA); Anexelekto (AXL).

In conclusion, the molecular mechanism of COVID-19-induced EMT is presented in Figure 2.

## 4 Molecular mechanisms of COVID-19-induced PF

### 4.1 ACE2-related PF by SARS-CoV-2 infection

Infection with SARS-CoV-2 can directly affect the expression of host cell surface receptors and signaling pathways and the production of cytokines. ACE2 acts as a receptor for SARS-CoV-2 infection and a regulator of the renin-angiotensin system (RAS) (McDonald, 2021). RAS plays an important role in maintaining hemostatic balance and fluid homeostasis by regulating the balance of ACE and ACE2 activities. By cleaving angiotensin 1 (Ang-1), ACE produces Ang-2, which activates a wide range of signaling pathways. ACE2/Ang-(1–7)/MasR axis, as one of the branches of RAS, plays an important role in anti-inflammation and anti-fibrosis (Delpino and Quarleri, 2020; Krygier et al., 2022). When SARS-CoV-2 binds to ACE2 receptors on the cell membrane of type II lung cells, ACE2 receptors of host cells undergo endocytosis, which reduces the expression of ACE2 and leads to the ability decrease of the host to balance RAS, thereby inhibiting the activity of ACE2/Ang-(1–7)/MasR axis, leading to pro-inflammation and pro-fibrosis (Krygier et al., 2022). The former mainly includes the activation of IL-6 and TNF-α as well as the increased recruitment of neutrophils and macrophages, while the latter mainly involves two key factors, MAPK/ERK and TGF-β (Vianello et al., 2022). Meanwhile, with the decrease of Ang-(1, 7) peptide, the concentration of TGF-β gradually increased, and played an important role in the process of fibrosis by promoting the formation of myofibroblasts from fibroblasts and collagen synthesis (Ni et al., 2020; Wigén et al., 2020; Vaz et al., 2021). Thus, the mechanism by which SARS-CoV-2 infects and enters host cells could directly activate host cell proinflammatory and profibrotic pathways. Furthermore, in IPF patients, ACE2 expression is increased in fibroblasts of PF tissue, suggesting that patients with PF are more susceptible to viral infection (Shen et al., 2021).

### 4.2 Role of adaptive immune cells in SARS-CoV-2 infection and PF

The adaptive immune system is extensively involved in the control of most viral infections and is composed of three main cell types: CD4^+^ T cells, CD8^+^ T cells and B cells. CD4^+^ T cells have a range of helper functions, capable of instructing B cells, assisting CD8^+^ T cells, and recruiting innate cells. Additionally, CD4^+^ T cells can also differentiate into effector cells such as Th1 cells, which have direct antiviral activity by producing IFN-γ and related cytokines (Sette and Crotty, 2021). In contrast, due to their ability to kill infected cells, CD8^+^ T cells play a crucial role in clearing various viral infections. In acute COVID-19, SARS-CoV-2-specific CD8^+^ T cells showed high levels of molecules, such as IFN-γ and granzyme B, which are associated with potent cytotoxic effector functions (Sette and Crotty, 2021). Studies have indicated that the presence of SARS-CoV-2-specific CD8^+^ T cells is correlated with better COVID-19 outcomes (Peng Y. et al., 2020; Rydyznski et al., 2020). Besides, B cells are the source of antibodies. In most COVID-19 patients, neutralizing antibodies develop rapidly within the same time frame as seroconversion. Neutralizing antibodies are produced by B cells, which possess extensive heavy and light chain V genes (Robbiani et al., 2020). Indeed, these adaptive immune cells play a significant role in the process of SARS-CoV-2 infection and subsequent PF. Among them, SARS-CoV-2-specific CD4^+^ T cells were most strongly associated with reduced disease severity in COVID-19 (Rydyznski et al., 2020). Mechanically, IL-22 has been reported to be robustly expressed by SARS-CoV-2-specific CD4^+^ T cells, and IL-22 is closely related to tissue repair, especially in lung epithelial cells, suggesting that SARS-CoV-2 CD4^+^ T cell response may play an active role in lung tissue repair during COVID-19. Moreover, as a subset of CD4^+^ T cells, Treg cells are mainly involved in the maintenance of immune tolerance (Zhang et al., 2021). Under the stimulation of inflammation, dendritic cell-derived IL-6 can induce the transformation of Treg cells to Th-17 cells. As a representative of the pro-inflammatory subset of CD4^+^ T cells, Th-17 cells primarily secrete the proinflammatory factor IL-17, which can increase the proliferation of fibroblasts and the production of collagen (Park et al., 2005; John et al., 2021). Furthermore, both Th-17 and Treg cells share a common key regulator TGF-β, which possesses the potential to promote fibrosis (Zhang et al., 2021). Of note, in COVID-19 patients with pneumonia, the ability of CD4^+^ T cells to produce IL-17 *in vitro* is increased, which can enhance inflammatory response and activate neutrophils, suggesting that T cell activation in COVID-19 patients is significantly biased toward Th-17 functional phenotype (De Biasi et al., 2020). Similarly, it has been reported that the frequency of Treg cells is reduced in patients with severe COVID-19. The low numbers of Treg cells and the increased numbers of Th-17 cells, leading to a decrease in the ratio of Treg/Th17 cells (Xu J. et al., 2020; Qin et al., 2020; Wang et al., 2020; Wu and Yang, 2020). The deviation of Treg/Th-17 cells toward Th-17 cells balance may support the pathogenesis of COVID-19 and lead to the massive release of proinflammatory cytokines and chemokines in COVID-19 patients, enhancing cytokine storm and promoting PF and ARDS (Jafarzadeh et al., 2021).

### 4.3 Macrophages and COVID-19-induced PF

Macrophages also play a crucial role in the process of COVID-19-induced PF. The underlying mechanism may be that lung macrophages are polarized into M1 macrophages under the induction of INF-γ and TNF-α secreted by Th-1. Meanwhile, they secrete IL-12 and induce proinflammatory active substances such as TNF-α, IL-6, and nitric oxide synthase (NOS), which promote the proliferation of fibroblasts, secretion of collagen, and aggravate PF (Cao et al., 2022). Notably, SARS-CoV-2 induced similar differentiation of classical monocytes *in vitro*, confirming that infection with the virus may be an inducer of profibrotic macrophage reprogramming. Some studies have explored the profibrotic characteristics of pulmonary macrophages in severe COVID-19 patients, and found severe fibrotic lung tissue remodeling (Wendisch et al., 2021). Besides, SARS-CoV-2 transcripts were detected in pulmonary macrophages, indicating that SARS-CoV-2 may directly induce the profibrotic macrophage phenotype. In several studies, SARS-CoV-2 infection induced the expression of fibrosis-related genes in macrophages, such as TGF-β1, secreted phosphoprotein 1 (SPP-1), and CCL18. These genes directly or indirectly contribute to the profibrotic function of macrophages (Bhattacharya, 2022). Furthermore, in the comparison of mass cytometry analysis of COVID-19 and control lung monocytes and macrophages, higher concentrations of IL-1β (a profibrotic factor) were observed in COVID-19 samples. Interestingly, macrophages are able to promote fibrosis through IL-1β and CCL18 synthesis (Oatis et al., 2022).

### 4.4 Galectin-3 and COVID-19-induced PF

Galectin-3 (Gal-3) is a member of the Galectin family and an important β-galactosidase-binding lectin. At the same time, it is the most studied possible therapeutic target of COVID-19 (Caniglia et al., 2020; Oatis et al., 2022). Due to its structural similarity to the N-terminal domain of coronavirus spike protein subunit 1 and its ability to bind ACE2 receptor, Gal-3 is widely involved in SARS-CoV-2 infection (Behloul et al., 2020). In addition, Gal-3 plays an important role in immune response, macrophage-associated cytokine storm, and COVID-19-induced PF (Caniglia et al., 2020; Garcia-Revilla and Caballero-Castillo, 2020). Importantly, Gal-3 is an important mediator of TGF-β-induced PF and can induce EMT and ECM production in PF by promoting TGF-β1 signaling (Sureshbabu et al., 2011; Mackinnon et al., 2012; Oatis et al., 2022). Studies have shown that Gal-3 is elevated in proliferative T lymphocytes associated with severe COVID-19 patients (Liao et al., 2020). Moreover, Gal-3 was one of the most upregulated genes associated with macrophage subsets expressing two fibrosis-related markers, triggering receptor expressed on myeloid cells 2 (TREM 2) and SPP-1, and both of them are involved in PF (Ramachandran et al., 2019; Garcia-Revilla and Caballero-Castillo, 2020; Liao et al., 2020). Infection with SARS-CoV-2 is often accompanied by a strong inflammatory response. Mechanistically, SARS-CoV-2 can activate NLRP3 inflammasomes through Gal-3, which can also control the release of proinflammatory cytokines such as IL-1, IL-6, TNF-α, and IL-1β (Boza-Serrano et al., 2019; Shi et al., 2019; Garcia-Revilla and Caballero-Castillo, 2020). Furthermore, the plasma level of Gal-3 is significantly correlated with the progression of PF, and the plasma level of Gal-3 is increased in COVID-19 patients, which may be related to the involvement of severe COVID-19 cytokine storm (Ho et al., 2016; De Biasi et al., 2020). Therefore, plasma Gal-3 levels are expected to play a vital role in the prognosis of COVID-19 inflammation.

### 4.5 Heat shock protein 90 and COVID-19-induced PF

Heat shock protein 90 (HSP90) is one of the most abundantly expressed chaperones involved in the stabilization and activation of more than two hundred proteins (Yadav et al., 2022; Zhao et al., 2023). HSP90 is known to regulate the viral life cycle by chaperoning various viral proteins, including SARS-CoV-2 (Kasperkiewicz and Tukaj, 2022). Previous studies have found that HSP90 is overexpressed in damaged lungs of COVID-19 patients, and HSP90 inhibitors can reduce SARS-CoV-2 infection, replication and the expression of proinflammatory cytokines (Barone et al., 2021; Wyler et al., 2021). Of note, a rising amount of literature have shown that HSP90 is involved in the formation and development of fibrosis, and is associated with increased inflammatory activity and deterioration of lung function, suggesting that HSP90 plays an important role in COVID-19-induced PF (Colunga et al., 2020). Specifically, HSP90 can critically affect the function of TGF-β by stabilizing the signaling cascade of TGF-β and promoting the folding and preservation of TGF-β receptors. On the one hand, HSP90 affects the TGF-β-dependent Smad signaling cascade by regulating the nuclear localization of Smad (Lee J. et al., 2016). On the other hand, HSP90 is able to promote AKT phosphorylation in the ERK signaling pathway, which is a downstream non-Smad signaling pathway of TGF-β1 signaling (Dong et al., 2017). In addition, HSP90 can also promote EMT metastasis and formation, and thus is considered an important regulator of EMT. It has been demonstrated that EMT and the activation of fibroblasts can be hindered by interfering with the binding of HSP90 and TGF-β receptor II, suggesting that EMT and the activation and of pulmonary fibroblasts induced by TGF-β1 can be attenuated by targeting HSP90 (Li et al., 2020). Furthermore, HSP90 has been reported to be involved in macrophage activation (Saha et al., 2018). Mechanistically, HSP90 inhibitor can attenuate the proinflammatory activity of M1 macrophages by inhibiting the p38 MAPK pathway in intervertebral disc degeneration. HSP90 has also been reported to be essential for the transactivation of iNOS (M1 marker) gene (Luo et al., 2011). Therefore, HSP90 has the potential to be a therapeutic target for COVID-19-induced PF. Many HSP90 inhibitors have been initially studied in the treatment of PF and deserve further attention.

### 4.6 SARS-CoV-2 activates proinflammatory pathways and induces PF

SARS-CoV-2 infection typically elicits an overreaction of the immune system, inducing a specific pattern of inflammation that is distinct from other viral infections (Vianello et al., 2022). This may lead to increased recruitment of inflammatory cells such as macrophages, neutrophils and monocytes, which may result in the release of numerous cytokines, as well as epithelial and endothelial cell lesions in the airways, lung tissue damage, and pulmonary cell infiltration (Darif et al., 2021; Saifi et al., 2022; Vianello et al., 2022). In severe cases, it can lead to lung tissue remodeling and PF (Schmitt et al., 2022). In detail, after virus entry into the epithelial cells of the respiratory tract, Th-1 are activated, which stimulate the production of proinflammatory cytokines such as GM-CSF and IL-6 (Krygier et al., 2022). In response to GM-CSF stimulation, monocytes produce a large number of cytokines such as IL-1β, IL-6, IL-7, IL-8, IL-9, IL-10, and TNF-α, which is called cytokine storm (Hu et al., 2021; Vianello et al., 2022). Moreover, substantial evidence has shown that elevated levels of proinflammatory cytokines in serum are positively associated with COVID-19 severity and mortality (Darif et al., 2021; Hu et al., 2021). Notably, among cytokines, IL-6, TNF-α and IL-1β have been identified as the key targets of COVID-19-induced PF. As one of the most important pro-inflammatory factors, IL-6 was significantly increased in the serum of COVID-19 patients compared with healthy people. In the study by Colarusso et al., characteristic changes in cytokine profiles were found that may contribute to PF. Compared with patients with COVID-19 but without fibrosis, the levels of IL-1β and TGF-α in post-COVID-19 patients with fibrosis were higher (Colarusso et al., 2021). The following approaches contribute to understanding the links between proinflammatory factors and other signaling pathways.

WNT signaling pathway plays a key role during COVID-19-induced PF and inflammation. The main functions of WNT signaling are to induce epithelial cell proliferation, EMT, myofibroblast differentiation and collagen synthesis (Chanda et al., 2019). The classical transmission pathways include WNT/β-catenin, WNT/PCP and WNT/Ca^2+^ pathways (Niehrs and Acebron, 2012; Hirawat et al., 2023). Amongst these pathways, WNT/β-catenin pathway mainly affects the inflammatory process and contributes to the activation of fibroblasts (Nusse and Clevers, 2017). For example, activation of the WNT/β-catenin pathway in AEC2 increases IL-1β production, which results in an inflammatory and profibrotic response. TGF-β1 has been shown to induce EMT by cooperating with WNT/β-catenin signaling pathway, which increases the risk of PF (Shen et al., 2020). In some clinical investigations of COVID-19 survivors, the presence of TGF-β was found to be correlated with the upregulation of WNT signaling pathway (Hirawat et al., 2023). At the same time, evidence has shown that WNT/β-catenin pathway is related to cytokine storm syndrome, and cytokine storm syndrome is also an important factor leading to ARDS and PF in COVID-19 patients (Choi et al., 2020). Besides, since WNT/β-catenin signaling is associated with taste, the loss of smell and taste observed in some COVID-19 patients suggests that WNT/β-catenin may be directly involved in COVID-19 infection (Saifi et al., 2022).

The Notch pathway has been well documented for its role in promoting inflammation (Breikaa and Lilly, 2021). Firstly, the Notch pathway is involved in macrophage polarization by promoting the M1 phenotype of macrophages over the M2 phenotype, thereby contributing to the expansion of the inflammatory loop (Keewan and Naser, 2020). Moreover, Notch 1 is able to directly bind to the IL-6 promoter in response to INF-γ in macrophages, thereby activating IL-6 transcription (Zhang Z. et al., 2020; Breikaa and Lilly, 2021). More importantly, IL-6, in turn, increases the expression of the Notch ligand Dll 1, further amplifying Notch signaling to produce more IL-6 (Breikaa and Lilly, 2021). Furthermore, Notch signaling also triggers inducible nitric oxide synthase (iNOS, whose expression has been implicated in cytokine storm) and other cytokines such as IL-4, and IL-13, and TNF-α (Baindara et al., 2022). These results all indicate multiple pathways by which Notch signaling drives hyperinflammation in COVID-19 infection.

**FIGURE 3:**
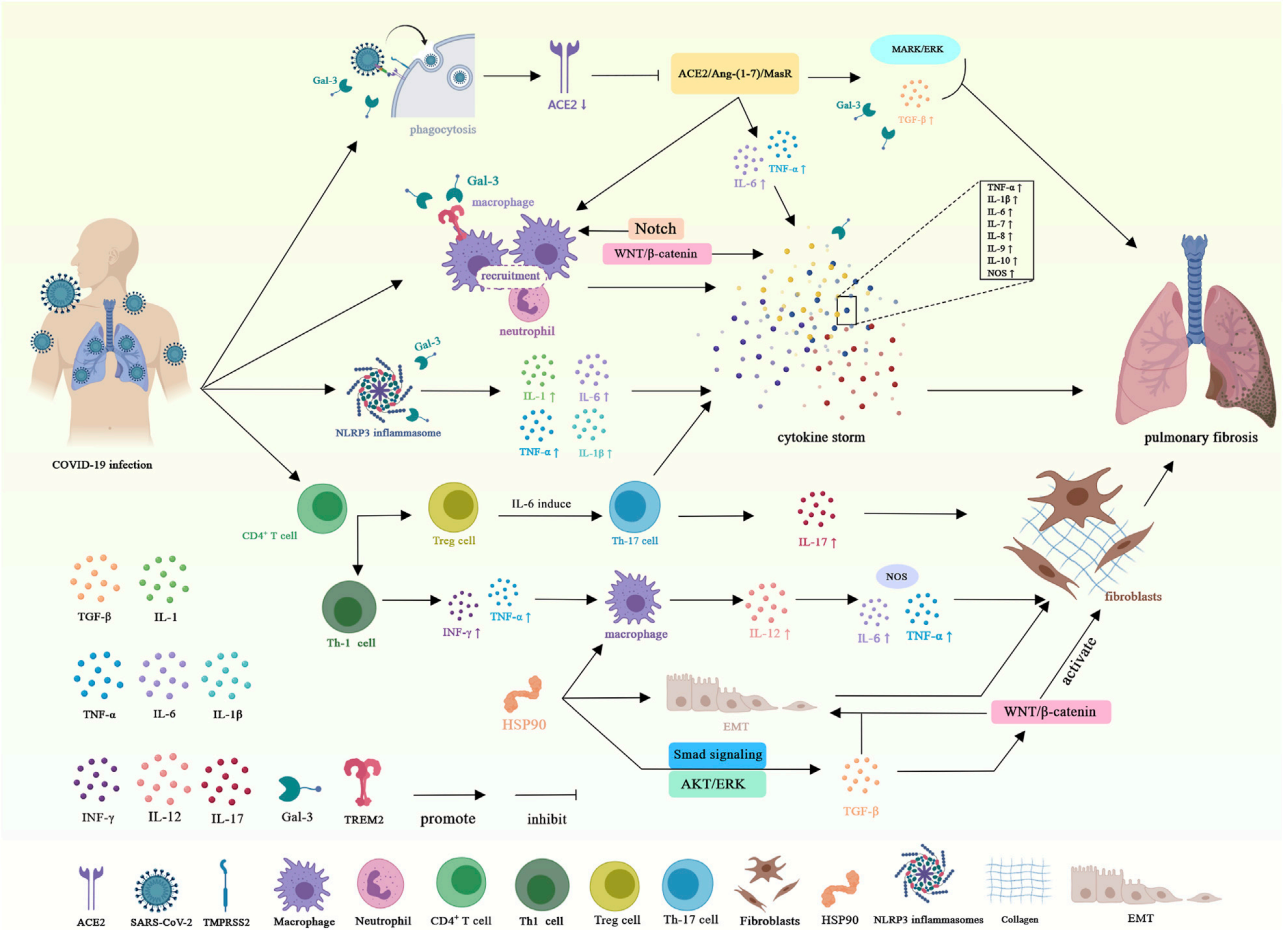
Schematic representation of the molecular mechanism of coronavirus-induced pulmonary fibrosis. Angiotensin-converting enzyme 2 (ACE2); Transmembrane serine protease 2 (TMPRSS2); Inter-leukin-1 (IL-1); Interleukin-1β (IL-1β); Tumor necrosis factor α (TNF-α); Transforming growth factor-β (TGF-β); Interleukin-6 (IL-6); Interferon γ (INF-γ); Interleukin-12 (IL-12); Interleukin-17 (IL-17); Galectin-3 (Gal-3); Triggering receptor expressed on myeloid cells 2 (TREM 2); Nucleotide-binding oligomerization domain-like receptor family pyrin domain-containing 3 (NLRP3); T helper 1 cell (Th-1); T helper 17 cell (Th-17); Nitric oxide synthase (NOS); HSP90 (Heat shock protein 90); Epithelial-mesenchymal transition (EMT).

In summary, the molecular mechanism of COVID-19-induced PF is shown in Figure 3.

## 5 Therapeutic options for COVID-19-induced PF

Even though multiple drugs, technologies, and vaccines are in clinical trials or clinical use for the treatment and prevention of COVID-19-induced PF, only nintedanib and pirfenidone are currently approved for the treatment of PF. In addition, some therapeutic substances have been shown to be effective in research, while the mechanism of action of others remains unclear or controversial, and some have only been shown to be effective in animal experiments. Here, we summarize these therapeutic substances and detailed mechanisms of action, targets, and indications are shown in Table 1.

**TABLE 1:** Potential therapeutic options for COVID-19-induced pulmonary fibrosis.

Therapeutic substance	Indication	Therapeutic target	Mechanism	Study progress	References
Nintedanib	COVID-19-induced PF	EGFR, FGFR, PDGFR, and VEGFR	Reduces the expression of ACE2 and the body’s response to SARS-CoV-2	Confirmed	Shen et al. (2021)
					Umemura et al. (2021)
Natalizumab	COVID-19-induced PF	Integrins	Inhibits integrin signaling, and virus entry	Unclear	Sigrist et al. (2020)
					Saifi et al. (2022)
Pirfenidone	COVID-19-induced PF	Serum and lung IL-6 levels	Inhibits lung damage, and reduces serum and lung IL-6 levels	Confirmed	Zhang et al. (2020a)
					Shen et al. (2021)
Poly-(ADP-Ribose) Polymerase inhibitor	COVID-19-induced PF and inflammation	Cytokine storms	Prevents cytokine storm (macrophage hyperactivation)	Confirmed by animal models	Curtin et al. (2020)
Propolis	COVID-19-induced PF and inflammation	Propolis block kinase PAK-1	Blocks the kinase PAK-1, and reduces the excessive inflammatory response	Confirmed	Berretta et al. (2020)
					Nile et al. (2020)
Spironolactone	COVID-19-induced PF	ACE2, mineralocorticoid receptors, TMPRSS2	Increases the circulating level of ACE2, prevents SARS-COV-2 entry, blocks the mineralocorticoid receptors, Downregulates TMPRSS2	Confirmed	Cadegiani et al. (2020)
					Kotfis et al. (2021)
Tocilizumab	COVID-19-induced PF	IL-6	Inhibits IL-6	Unclear	Gautret et al. (2020)
Tocilizumab	PF complicated with COVID-19	Immunomodulatory effects	Inhibits pulmonary fibrosis and prevents cytokine storms	Confirmed	Gatti et al. (2021)
Treamid	COVID-19-induced PF	Inflammation	Suppresses inflammation and restores the diffusing capacity of the lungs	Unclear	Skurikhin et al. (2020)
					Bazdyrev et al. (2021)
Histone deacetylase inhibitors (TGF-β1 inhibitors)	Post COVID-19-induced PF	TGF-β1 signaling	Inhibits TGF-β1 signaling pathway	Confirmed	P et al. (2021)
CD147 inhibitors	COVID-19-induced PF	CD147 receptor, TGF-β1 signaling	Inhibits TGF-β1-induced proliferation and differentiation of fibroblasts into myofibroblasts	Confirmed	Ulrich and Pillat (2020)
Corticosteroids	Post COVID-19-induced PF	Caveolin-1, TNF-a, TGF-β1, PDGF	Elevates caveolin-1 levels, reduces TNF-α, TGF-β1, and PDGF levels, decreases inflammation in the lungs	Confirmed in animal studies	Bazdyrev et al. (2021)
Collagen-Polyvinylpyrrolidone	COVID-19-induced PF	Cytokine storms	Decreases IL-1β, IL-8, TNF-α, TGF-β1, IL-17, Cox-1, leukocyte adhesion molecule (ELAM-1, VCAM-1 and ICAM-1) levels, reduces the expression of other inflammatory mediators, increases IL-10 level, and the amount of Treg cells	Unclear	Bazdyrev et al. (2021)
Galectin-3 inhibitor	COVID-19-induced PF	Galectin-3	Prevents Gal 3 from binding and activating TLR 4 and TREM	Unclear	Shen et al. (2021)
Genistein	COVID-19-induced PF	NF-κB	Inactivates NF-κB	Unclear	Bazdyrev et al. (2021)
Anakinra	Post COVID-19-induced PF	IL-1, IL-6	Inhibits IL-1 and IL-6	Confirmed	George et al. (2020)
Deupirfenidon	COVID-19-induced PF	Inflammation	Anti-inflammatory and antifibrotic activity	Unclear	Bazdyrev et al. (2021)
Fuzheng Huayu	Post COVID-19-induced PF	Matrix metalloproteinase 2, type IV collagen	Suppresses the activity of matrix metal-loproteinase 2, and type IV collagen expression	Unclear	Bazdyrev et al. (2021)
IN01 Vaccine	COVID-19-induced PF	EGF, EGFR	Inhibits the binding of EGF to its receptor, blocks EGFR activation as well	Unclear	Bazdyrev et al. (2021)

PF, pulmonary fibrosis; EGFR, epidermal growth factor receptor; ACE2, angiotensin-converting enzyme 2; IL-6, interleukin-6; PAK-1, p21-activated protein kinase-1; TMPRSS2, transmembrane serine protease 2; TNF-α, tumor necrosis factor α; TGF-β1, transforming growth factor-β1; PDGF, platelet-derived growth factor; IL-1β, interleukin-1β; IL-8, interleukin-8; IL-17, interleukin-17; ELAM-1, endothelial leukocyte adhesion molecule 1; VCAM-1, vascular cellular adhesion molecule-1; ICAM-1, intercellular adhesion molecule 1; IL-10, interleukin-10; Gal 3, galectin 3; TLR, 4 = toll-like receptor-4; TREM, triggering receptor expressed on myeloid cells; NF-κB, nucleus factor-κB; IL-1, interleukin-1; EGF, epidermal growth factor; FGFR, fibroblast growth factor receptor; PDGFR, platelet-derived growth factor receptor; VEGFR, vascular endothelial growth factor receptor.

## 6 Future perspective

Although many studies have proposed the pathogenesis of COVID-19-induced PF, it can be divided into the following main commonalities: 1) Viral infection leads to abnormal expression of profibrotic TGF-β and induces PF. 2) At the same time, the lung is damaged by virus infection, which causes abnormal immune response. For example, immune cells release large amounts of proinflammatory and profibrotic cytokines/factors. 3) Viral infection directly or indirectly drives EMT to induce PF. 4) Viral infection resulted in decreased expression of ACE2, which shifted the balance of RAS towards profibrotic direction. At present, the molecular mechanism of COVID-19-induced PF and EMT is still in the stage of speculation. The molecular mechanisms underlying the COVID-19-induced profibrotic genes still needs to be studied in more detail. Comparing PF in COVID-19 with other types of viral PF may reveal different features and mechanisms.

It is also worth noting that despite the specific evidence of fibrosis development in COVID-19 patients and residual fibrosis in COVID-19 survivors, fibrosis is a normal repair process that is almost inevitably accompanied by other tissue changes and inflammatory responses. Whether to treat COVID-19-induced fibrosis with antifibrosis remains unknown, given that COVID-19-related fibrosis-like reactions are self-resolving in nature and PF seen in patients with COVID-19 resolves over time. In addition, because fibrosis is generally considered to be an irreversible process, drug therapy mainly delays functional decline and cannot completely cure fibrosis, which greatly limits the use of drugs. Since SARS-CoV-2 can also cause long-term lung injury and persistent PF, timely antiviral and antifibrosis treatment is still necessary. The safety of traditional anti-fibrosis drugs and the efficacy of new therapeutic substances need to be further studied. In addition, there are few drug studies on COVID-19-driven EMT as a therapeutic target, considering that EMT is one of the important pathways to induce PF, which may be a good choice for future drug treatment studies.

## 7 Conclusion

Much has yet to learn and concern about the long-term effects of COVID-19, as well as other comorbidities. This review highlights the importance of COVID-19-induced EMT and PF in COVID-19 patients, and summarizes the molecular mechanisms of its pathogenesis and potential treatment. Although there are still many uncertainties in the use of antifibrotic drugs in patients with COVID-19, with the advancement of more and more clinical trials and research, the relevant pathological mechanisms may become clearer. Timely management and regulation of fibrosis in patients with COVID-19 may prevent the long-term effects of fibrosis after COVID-19 and improve the quality of life of patients.
